# Oral Health Impact Profile in Orthodontic Patients with Ectopic Canine: A Prospective Clinical Intervention of Four Treatment Modalities

**DOI:** 10.3390/healthcare9030337

**Published:** 2021-03-16

**Authors:** Mohammad Khursheed Alam, Ahmed Ali Alfawzan

**Affiliations:** 1 Orthodontic Division, Department of Preventive Dentistry, College of Dentistry, Jouf University, Sakaka 72345, Saudi Arabia; 2 Department of Preventive Dentistry, College of Dentistry in Ar Rass, Qassim University, Ar Rass 58876, Saudi Arabia; ah.alfawzan@qu.edu.sa

**Keywords:** OHRQoL, OHIP-16, ectopic canine, crowding, orthodontic treatment modalities

## Abstract

This study evaluated the oral health-related quality of life (OHRQoL) in Saudi subjects with ectopic canine, severe crowding, and class I normal occlusion. Moreover, we assessed the differences among orthodontic treatment modalities (OTM) in the ectopic canine group. Study design: Retrospective and prospective evaluation. A total of 96 subjects were assessed for OHRQoL using the Oral Health Impact Profile, English version (OHIP-16). Initial comparison of the baseline data of ectopic canine subjects was made with severe crowding (>8 mm) and class I normal occlusion with the matched number and age of the subjects in later groups. Moreover, a comparison of OHIP-16 scores among four OTM in the ectopic canine group, baseline, 1 day, and 28 days after treatment were performed. OHIP-16 scores of ectopic canine, severe crowding, and class I normal occlusion group were 33.06, 33.09, and 26.43, respectively. Subjects with ectopic canine and severe crowding reported a significantly greater negative impact in terms of embarrassment, avoiding smiling, and lack of self-confidence. OHRQoL had no significant changes among the four OTM groups (*p* > 0.05). Untreated malocclusion had a significant negative impact on OHRQoL. The negative impact was seen in several domains 1 day after treatment, and gradual improvement was noted 28 days after treatment.

## 1. Introduction

Subjects with orthodontic needs approach a clinician primarily to address their dentofacial aesthetics and to improve their oral health [1]. However, as in other interventions, treatment with the fixed orthodontic appliances is not free of associated complications or risks. Pain, mobility, delayed tooth movement, root resorption, and loss of vitality at times may be some of the associated untoward effects of the treatment, which could be due to excessive force applied for orthodontic tooth movement [2]. In addition, due to difficulty in brushing, there may be accumulation of plaque around the brackets, resulting in halitosis, decalcification of tooth/teeth surfaces, gingival inflammation, and periodontal disease [3].

Correction of malposed teeth, discrepancies related to the jaw growth, and their relation are assessed by an orthodontist since inception. Orthodontic treatment modalities (OTM) have a positive effect on functional demands, dental health, and aesthetics. Such treatment has a significant effect on the community, corporeal, and psychological comfort [4,5]. However, it is also associated with deleterious effects that have an impact on the quality of life. Hence, a significant drive for the assessment of oral health-related quality of life (OHRQoL) in orthodontics is gaining momentum the world over [6,7].

Every tooth, as it erupts from its developmental position within the jaws to its functional position in the occlusal plane, erupts into a specific, predetermined position in the dental arch. However, sometimes there is a deviation in its path of eruption, and such teeth erupt in abnormal locations, which are termed as ectopic eruption. According to Nikiforuk (1948), ectopic eruption is a condition where the permanent tooth takes an abnormal path of eruption due to deficiency in the jaw growth or a segment of jaw and encounters a deciduous tooth, leading to its premature exfoliation and malposition of the permanent tooth [8].

The maxillary and mandibular canines are anterior teeth that are placed at the corners of the mouth, and their positions and forms of these teeth along with the canine eminence play a vital role in aesthetics and face value. The inclination of the upper canine also influences the aesthetics specially a smile [9]. Hence, any change in its position or early loss may not only unduly affect the general appearance but may also psychologically impact an individual.

The underlying concepts of various OTM in therapeutic orthodontics are simple and easily understood. However, the type, degree of malocclusion, treatment stages, and the choice of treatment modalities may affect the treatment duration. It can be assumed that the impact of ectopically placed canine can affect the psychological development of the child/individual and may affect in general the quality of life. Early diagnosis of ectopic maxillary canines and the corrective measures would minimize the negative impact and boost up the morale and confidence of the individuals affected.

Studies revealed that low level laser therapy (LLLT) has promising benefits on osteogenesis in vitro [10,11], on bone remodeling during orthodontic tooth movement in rats [12], on acceleration of orthodontic tooth movement in animals [13] and humans [14,15], on spontaneous and chewing pain caused by elastomeric separators [16], and on pain associated with the initial stage of fixed orthodontic treatment [17,18]. However, LLLT as OTM on OHRQoL has never been investigated.

This first-in-human study compared OHRQoL using Oral Health Impact Profile, English version (OHIP-16) scores among ectopic canine, severe crowding, and class I normal occlusion subjects’ group and the changes in OHRQoL using OHIP-16 scores [19], baseline, 1 day, and 28 day following insertion of fixed orthodontic appliances in four different OTM groups in ectopic canine subjects.

## 2. Materials and Methods

Ninety-six (96) healthy subjects of Saudi ethnic background, aged between 14 and 25 years, were selected for the study. Thirty-two (32) subjects in the ectopic canine group, on the basis of OTM, were randomly divided into 4 groups, namely, LLLT + (self-ligating bracket) SLB, non-LLLT + SLB, LLLT + conventional bracket (Conv.), and non-LLLT + Conv. group. Inclusion, exclusion criteria, study groups, 4 different OTM, number of subjects in each group, armamentarium used, orthodontic treatment, laser application, OHIP-16 assessment [19], ethical aspects, and statistical analysis are detailed in Table 1.

For initial comparison, OHIP-16 data of the study subjects were collected. OHIP-16 score among 3 groups (ectopic canine, severe crowding (>8 mm crowing), and class I normal occlusion (class I molar, incisor and canine relation, without any crowding, normal overjet and overbite, without any orthodontic treatment)) were analyzed using one-way ANOVA to see the differences. Later, OHIP-16 baseline data of the ectopic canine group was compared using repeated-measure ANOVA with the OHIP-16 data of 1 day and 28 days after commencement of the 4 different OTM.

## 3. Results

Among seven domains of OHIP-16 scores, psychological discomfort (embarrassment, *p* ≥ 0.001), physical disability (avoid smiling, *p* ≥ 0.001), and handicap (lack of self-confidence, *p* ≥ 0.001) showed highly significant disparities among the three groups (Table 2). Total OHIP-16 scores of ectopic canines and severe crowding subjects’ group was comparatively higher than class I normal occlusion group, however, no significant disparities were found among the three groups (Table 2).

Table 3 shows mean ± SD values of all seven domains of OHIP-16 scores in ectopic canine subjects’ group among the four different OTM (baseline, 1 day, and 28 days after fixed orthodontic treatment).

**Figure 1:**
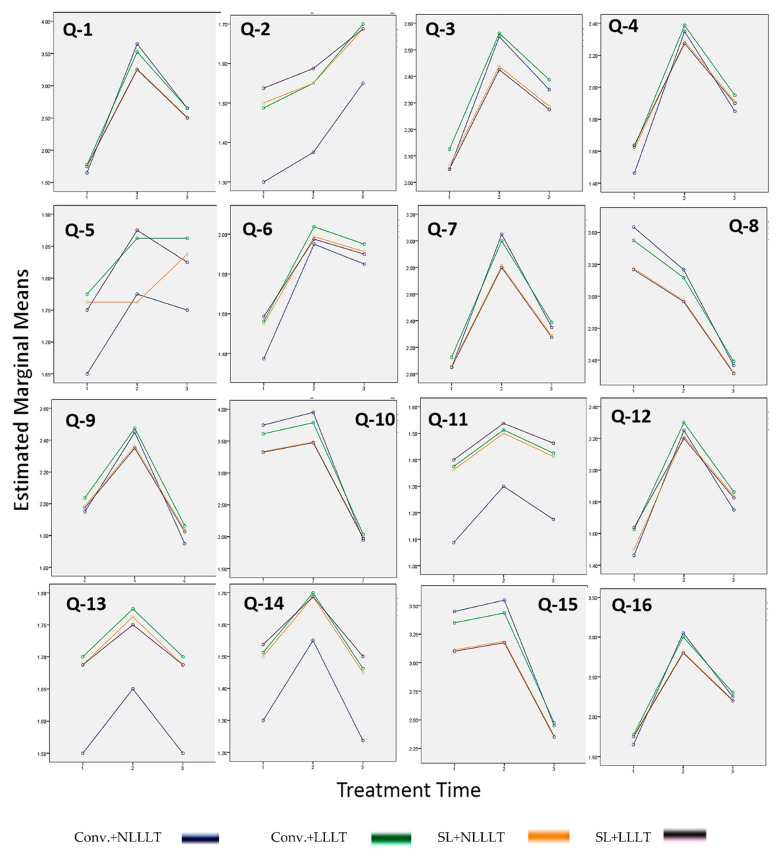
Factor plot charts of OHIP-16 scores of four different OTM groups. Treatment time: 1 = baseline, 2 = 1 day after bonding, and 3 = 28 days after bonding.

Figure 1 shows the factor plot charts of OHIP-16 scores of four different OTM groups (baseline, 1 day, and 28 days after fixed orthodontic treatment). No significant disparities were found in all OHIP-16 scores among the four OTM groups. However, treatment time duration (gradual improvement of OHRQoL) showed significant changes in all OHIP-16 scores.

## 4. Discussion

Previous studies relating to malocclusion and orthodontic treatment with OHRQoL were found to be ambiguous. While few studies proved that there is a correlation with the orthodontic need or malocclusion and OHRQoL, some others indicated that there are no clear relationships [21,22]. In this study, statistical analysis showed that participants with severe malocclusions reported significantly greater negative impact on OHRQoL (Table 2). The varied findings of these studies could be due to differences in the use of measures, age groups, cultures, traditions, races and ethnic groups, and social norms across countries [21,22].

In the current study, embarrassment, avoiding smiling, and lack of self-confidence were significantly higher in the severe crowding group, followed by the ectopic canine group compared to the class I normal occlusion group. These findings suggest that they suffered from aesthetic and social issues. However, their daily activities remained unaffected and any small variations in orthodontic treatment needs that might significantly affect perceived OHRQoL in the psychological discomfort domain. de Oliveira et al., [23], O’Brien et al., [24], and Feu et al. [25] strongly suggest that there is a positive association between malocclusion and psychological discomfort. Questions relating to emotional and social domains including getting upset, embarrassment, shyness, and stopping smiling or laughing in the psychological scales are more applicable to orthodontic patients [26]. Secondly, the patients with greater orthodontic needs presented greater psychological impairment as compared to those with no or borderline treatment needs. According to Zhou et al. [27], about half of the patients with malposed teeth had nicknames associated with their dentofacial problems, and 80% of these patients were angry or upset with such nicknames. Moreover, it was shown by Zhou et al. [27] that the psychological status of patients with skeletal malocclusion was directly related to the severity of malocclusion.

Patients with malocclusion may get easily embarrassed in social contexts and their self-concept may be negatively affected [26,28]. Most of the patients seeking orthodontic treatment want their dental aesthetics to be corrected and improve self-esteem [25]. Thus, it is important that the orthodontist should plan the treatment to improve oral function and health, aesthetics, self-esteem, and social activities. As a part of the diagnostic procedure, collecting data pertaining to OHRQoL may indicate the priorities for the treatment to achieve maximum patient satisfaction [25]. However, the OHIP-16 has only four items (of 16) related to the psychological status evaluation. Liu et al. [29] have suggested that an ideal instrument with more considerations of psychological aspects for assessing OHRQoL research may be required as it may play significant role in evaluating quality of life in patients with malocclusion.

Irrespective of the reason, adverse effects of the OTM proportionate directly with the duration of the treatment. Currently, the duration of orthodontic treatment with fixed braces is 2 to 3 years on average [30]. However, the patient does not expect more than 1.5 years [31]. Prolonged treatment duration may also be deleterious efficiency of the national healthcare system and also private practices. Thus, reducing the duration of the treatment by accelerating the tooth movement has been a perennial concern for both orthodontists and patients [32]. OHRQoL in orthodontic patients essentially depends on these variables and outcome. The current research explored the OHRQoL on the basis of the four different OTM (laser and non-laser in Conv. and SLB), observed for baseline, 1 day, and 28 days after fixed orthodontic treatment. The findings suggest the OHRQoL, 1 day, and 28 days after orthodontic treatment regardless of different OTM has been changed and gradually improved with the duration. Drastic changes in all seven domains of OHIP-16 scores were revealed 1 day after orthodontic treatment. These findings are quite similar to those of Mansor et al. [19] in relation to 1 day after orthodontic treatment. OHIP-16 scores for baseline, 1 day, and 28 days after fixed orthodontic treatments of four different OTM showed no changes in OHRQoL in domain numbers 2 (physical pain), 5 (psychological disability), and 6 (social disability). Among OHIP-16, questions 1 (difficulties in chewing), 4 (discomfort in eating), 7 (food stuck in between teeth), and 9 (avoidance of eating certain foods) showed bad impact in OHRQoL 1 day after orthodontic treatment. However, the bad impact gradually reduced 28 days after orthodontic treatment. Furthermore, domain number 3 (psychological discomfort), question 8 (embarrassment), domain number 4 (physical disability), and question 10 (avoid smiling) showed good impact 28 days after orthodontic treatment.

This first-in-human study revealed some significant and non-significant results related to OIHP-16 scores. Three different group comparisons on OHRQoL were investigated. In the ectopic canine group, four different OTM with three different treatment time duration data comparisons were also assessed. However, there were some limitations that need to be overcome to reveal more robust results. Longitudinal follow-up with larger sample size is important. This study was performed in a single ethnic population, and future studies with different populations might reveal some disparities in OHRQoL. The researchers encourage a similar type of study in a different subject group, with different OTM and OHIP-16 scores after completion of the treatment and in a different population.

## 5. Conclusions

This first-in-human research explored the OHRQoL in ectopic canine, severe crowding, and class I normal occlusion subjects. Highly significant disparities were revealed among the three groups. This study also revealed, among four different OTM groups in ectopic canine subjects, that there were no significant disparities in OHIP-16 scores. For the baseline, 1 day, and 28 days after fixed orthodontic treatment, OHRQoL was significantly different.

## Figures and Tables

**Table 1:** Subjects and methodology in detail.

Selection Guideline	Inclusive Healthy Orthodontic Patients of Saudi Ethnic Background: Angle Class I or II or III Malocclusion with Ectopic Maxillary Canine Requiring Space Creation or Extraction of First Premolar			Exclusive Patient on Long-Term Medication, Craniofacial Anomalies/Malformation, with Parafunctional Habits, Temporomandibular Joint Dysfunction, Multiple Missing Teeth, and Periodontally Compromised	
Study design	Objective 1: Retrospective study, and objective 2: prospective evaluation				
Sample size calculation: Objective 1	Utilizing effect-size r 0.63 [20], using G*Power software version 3.0.10 with power 80%; α 0.05 the total sample size intended for this research was 96 for three groups. Thus, 32 subjects were required for each group.				
Study groups (baseline)	Ectopic canine = 32		Severe crowding = 32		Class I normal occlusion = 32
Study groups (ectopic canine)	Laser			Non-laser	
Sample size calculation: Objective 2	G*Power software version 3.0.10 with power 80%, α 0.05, and effect size (d) 0.22 was used. Hence, the total sample size intended for this research was 32 for four different treatment modalities [18], and thus each group required a minimum of eight subjects.				
Treatment modalities	Self-ligating	Conventional		Self-ligating	Conventional
Number of subjects	8	8		8	8
Armamentarium	The laser unit was a 940-nm aluminum–gallium–arsenide (Al–Ga–As) diode laser (iLase; Biolase, Irvine, CA, USA) set on continuous mode with power at 100 mW. The diameter of the optical fiber tip was 0.04 cm^2^, the energy density was calculated to be 7.5 J/cm^2^ for each point, and total energy density was 75 J per tooth.				
Orthodontic treatment	For all patients, treatment was commenced by bonding the upper arch with preadjusted edgewise 0.022-inch slot MBT prescription brackets, Agility self-ligating bracket system (G&H Orthodontics, Franklin, IN, USA), and Ortho Organizers conventional type bracket system (Carlsbad, CA, USA). Alignment and leveling started using 0.012-inch super-elastic nickel–titanium (NiTi) wire and was followed by 0.014, 0.016, and 0.018-in NiTi wires, changed at 4-week intervals between each wire.				
Laser application	Laser applied on gingival mucosa for 3 s each on five points labially/buccally and palatally per tooth, starting from central incisor to the first molar. These points were mesial and distal over the cervical-third of the root and middle of the root, and mesial and distal over the apical-third of the root. The fiber tip of the laser was in close but light contact with the surface of the gingival tissues and held perpendicular to the mucosa overlying the roots of teeth.				
OHIP-16	The OHIP-16 measures focus on the impact of one’s oral health condition on QoL, contributing to seven domains: functional limitation, physical pain, psychological discomfort, physical disability, psychological disability, social disability, and handicap. Responses of each item are made on a Likert scale and coded as 1 = never, 2 = hardly ever, 3 = occasionally, 4 = fairly often, and 5 = very often. The OHIP-16 scores range from 16 to 80, where 16 indicates no impact and 80 indicates the worst impact of one’s oral health on QoL. Individual domain scores can be calculated by summing responses to the items within a domain, with higher scores indicating greater impact.				
Data collection	Patients completed the first questionnaire, which was used as the baseline, before insertion of the fixed orthodontic appliance, and they completed the second and third questionnaire 24 h and 28 days after insertion, respectively. These questionnaires were given to the participants to be completed at home and returned at the following appointment. The participants were asked to record OHIP-16 score after 24 h and 28 days. In addition, telephone calls/message were made at day 28 to ensure accurate collection of data.				
Statistical analysis	IBM SPSS Statistics version 22.0 (IBM Co., Armonk, NY, USA) was used to analyze the data. Descriptive analysis was performed to obtain the mean values of OHIP-16 scores among four groups. One-way ANOVA and repeated measure ANOVA were used for comparison.				

**Table 2:** Oral Health Impact Profile, English version (OHIP-16) score comparison among ectopic canine, severe crowding, and class I normal malocclusion subjects.

Domain	Items	Groups	Mean	SD	95% CI		*p*
					Lower Bound	Upper Bound	
Functional limitation	Difficulties in chewing	Ectopic canine	1.81	0.41	1.66	1.96	0.34
		Malocclusion	1.80	0.44	1.43	2.17	
		Normal occlusion	2.05	0.47	1.66	2.44	
		Total	1.85	0.42	1.73	1.97	
	Bad breath	Ectopic canine	1.53	0.30	1.42	1.64	0.48
		Malocclusion	1.53	0.32	1.26	1.79	
		Normal occlusion	1.68	0.34	1.39	1.96	
		Total	1.55	0.30	1.47	1.64	
	Difficulties in pronunciation	Ectopic canine	2.15	0.49	1.97	2.32	0.24
		Malocclusion	2.14	0.54	1.69	2.59	
		Normal occlusion	1.83	0.38	1.51	2.14	
		Total	2.09	0.49	1.95	2.23	
	Discomfort in eating	Ectopic canine	1.66	0.35	1.53	1.79	0.97
		Malocclusion	1.65	0.39	1.33	1.97	
		Normal occlusion	1.69	0.32	1.42	1.96	
		Total	1.66	0.35	1.56	1.76	
Physical pain	Ulcer	Ectopic canine	1.81	0.41	1.66	1.96	0.93
		Malocclusion	1.80	0.44	1.43	2.17	
		Normal occlusion	1.75	0.36	1.45	2.05	
		Total	1.80	0.40	1.68	1.91	
	Pain	Ectopic canine	1.59	0.32	1.48	1.71	0.22
		Malocclusion	1.59	0.35	1.30	1.88	
		Normal occlusion	1.83	0.38	1.51	2.14	
		Total	1.63	0.34	1.53	1.73	
Psychological discomfort	Food stuck in between teeth	Ectopic canine	2.15	0.49	1.97	2.32	0.06
		Malocclusion	2.14	0.54	1.69	2.59	
		Normal occlusion	1.69	0.32	1.42	1.96	
		Total	2.07	0.50	1.92	2.21	
	Embarrassment	Ectopic canine	3.50	0.99	3.14	3.85	0.00
		Malocclusion	3.54	1.00	2.71	4.37	
		Normal occlusion	1.69	0.32	1.42	1.96	
		Total	3.20	1.13	2.87	3.53	
Physical disability	Avoidances of eating certain foods	Ectopic canine	2.06	0.47	1.89	2.23	0.23
		Malocclusion	2.05	0.52	1.62	2.48	
		Normal occlusion	1.75	0.36	1.45	2.05	
		Total	2.01	0.46	1.87	2.14	
	Avoid smiling	Ectopic canine	3.58	1.03	3.21	3.95	0.00
		Malocclusion	3.63	1.03	2.77	4.48	
		Normal occlusion	1.75	0.36	1.45	2.05	
		Total	3.28	1.16	2.95	3.62	
Psychological disability	Disturbed sleep	Ectopic canine	1.38	0.14	1.33	1.43	0.61
		Malocclusion	1.38	0.15	1.25	1.50	
		Normal occlusion	1.44	0.17	1.30	1.58	
		Total	1.39	0.15	1.35	1.43	
	Concentration affected	Ectopic canine	1.63	0.34	1.51	1.76	0.47
		Malocclusion	1.65	0.39	1.33	1.97	
		Normal occlusion	1.48	0.20	1.31	1.64	
		Total	1.61	0.33	1.51	1.70	
Social disability	Avoided going out	Ectopic canine	1.73	0.38	1.59	1.87	0.21
		Malocclusion	1.73	0.41	1.38	2.07	
		Normal occlusion	1.48	0.20	1.31	1.64	
		Total	1.69	0.37	1.58	1.79	
	Difficulty carrying out daily activities	Ectopic canine	1.54	0.29	1.43	1.64	0.85
		Malocclusion	1.53	0.32	1.26	1.79	
		Normal occlusion	1.48	0.20	1.31	1.64	
		Total	1.53	0.28	1.45	1.61	
Handicap	Lack of self-confidence	Ectopic canine	3.33	0.92	2.99	3.66	0.00
		Malocclusion	3.36	0.93	2.58	4.14	
		Normal occlusion	1.56	0.27	1.34	1.79	
		Total	3.04	1.07	2.73	3.35	
	Difficulties in cleaning	Ectopic canine	1.81	0.41	1.66	1.96	0.16
		Malocclusion	1.80	0.44	1.43	2.17	
		Normal occlusion	1.51	0.24	1.32	1.71	
		Total	1.76	0.40	1.64	1.87	
Total	OHIP-16	Ectopic canine	33.06	7.12	30.49	35.62	0.06
		Malocclusion	33.09	7.68	26.67	39.51	
		Normal occlusion	26.43	4.64	22.54	30.31	
		Total	31.96	7.19	29.87	34.04	

**Table 3:** OHIP-16 score comparison among ectopic canine subjects undergoing 4 different orthodontic treatment modalities (OTM), baseline, 1 day, and 28 days after orthodontic treatment.

Domain	Items	OTM	Baseline		1 Day after		28 Days after	
			Mean	SD	Mean	SD	Mean	SD
Functional limitation	Difficulties in chewing	Conv. + NLLLT	1.65	0.37	3.65	0.37	2.65	0.37
		Conv. + LLLT	1.78	0.43	3.53	0.95	2.65	0.64
		SL + NLLLT	1.76	0.36	3.26	1.18	2.51	0.74
		SL + LLLT	1.75	0.49	3.25	1.25	2.50	0.83
	Bad breath	Conv. + NLLLT	1.30	0.31	1.38	0.34	1.55	0.37
		Conv. + LLLT	1.49	0.34	1.55	0.36	1.70	0.40
		SL + NLLLT	1.50	0.24	1.55	0.28	1.69	0.34
		SL + LLLT	1.54	0.33	1.59	0.37	1.69	0.46
	Difficulties in pronunciation	Conv. + NLLLT	2.05	0.37	2.55	0.37	2.35	0.37
		Conv. + LLLT	2.13	0.49	2.56	0.62	2.39	0.56
		SL + NLLLT	2.06	0.50	2.44	0.70	2.29	0.62
		SL + LLLT	2.05	0.61	2.43	0.79	2.28	0.71
	Discomfort in eating	Conv. + NLLLT	1.46	0.35	2.35	0.37	1.85	0.37
		Conv. + LLLT	1.63	0.38	2.39	0.56	1.95	0.46
		SL + NLLLT	1.61	0.31	2.29	0.62	1.91	0.43
		SL + LLLT	1.64	0.41	2.28	0.71	1.90	0.55
Physical pain	Ulcer	Conv. + NLLLT	1.65	0.37	1.78	0.32	1.75	0.37
		Conv. + LLLT	1.78	0.43	1.86	0.46	1.86	0.44
		SL + NLLLT	1.76	0.36	1.76	0.36	1.84	0.39
		SL + LLLT	1.75	0.49	1.88	0.49	1.83	0.52
	Pain	Conv. + NLLLT	1.38	0.34	1.95	0.37	1.85	0.37
		Conv. + LLLT	1.56	0.35	2.04	0.47	1.95	0.46
		SL + NLLLT	1.55	0.28	1.99	0.46	1.91	0.43
		SL + LLLT	1.59	0.37	1.98	0.58	1.90	0.55
Psychological discomfort	Food stuck in between teeth	Conv. + NLLLT	2.05	0.37	3.05	0.37	2.35	0.37
		Conv. + LLLT	2.13	0.49	3.00	0.76	2.39	0.56
		SL + NLLLT	2.06	0.50	2.81	0.92	2.29	0.62
		SL + LLLT	2.05	0.61	2.80	0.99	2.28	0.71
	Embarrassment	Conv. + NLLLT	3.65	0.37	3.25	0.37	2.35	0.37
		Conv. + LLLT	3.53	0.95	3.18	0.82	2.39	0.56
		SL + NLLLT	3.26	1.18	2.96	1.01	2.29	0.62
		SL + LLLT	3.25	1.25	2.95	1.08	2.28	0.71
Physical disability	Avoidances of eating certain foods	Conv. + NLLLT	1.95	0.37	2.45	0.37	1.75	0.37
		Conv. + LLLT	2.04	0.47	2.48	0.59	1.86	0.44
		SL + NLLLT	1.99	0.46	2.36	0.66	1.84	0.39
		SL + LLLT	1.98	0.58	2.35	0.75	1.83	0.52
	Avoid smiling	Conv. + NLLLT	3.75	0.37	3.95	0.37	1.95	0.37
		Conv. + LLLT	3.61	0.98	3.79	1.04	2.04	0.47
		SL + NLLLT	3.34	1.23	3.49	1.32	1.99	0.46
		SL + LLLT	3.33	1.29	3.48	1.38	1.98	0.58
Psychological disability	Disturbed sleep	Conv. + NLLLT	1.09	0.15	1.30	0.31	1.18	0.23
		Conv. + LLLT	1.38	0.18	1.51	0.31	1.43	0.24
		SL + NLLLT	1.36	0.12	1.50	0.24	1.41	0.18
		SL + LLLT	1.40	0.16	1.54	0.33	1.46	0.24
	Concentration affected	Conv. + NLLLT	1.46	0.35	2.25	0.37	1.75	0.37
		Conv. + LLLT	1.63	0.38	2.30	0.54	1.86	0.44
		SL + NLLLT	1.50	0.22	2.21	0.58	1.84	0.39
		SL + LLLT	1.64	0.41	2.20	0.68	1.83	0.52
Social disability	Avoided going out	Conv. + NLLLT	1.55	0.37	1.65	0.37	1.55	0.37
		Conv. + LLLT	1.70	0.40	1.78	0.43	1.70	0.40
		SL + NLLLT	1.69	0.34	1.76	0.36	1.69	0.34
		SL + LLLT	1.69	0.46	1.75	0.49	1.69	0.46
	Difficulty carrying out daily activities	Conv. + NLLLT	1.30	0.31	1.55	0.37	1.24	0.27
		Conv. + LLLT	1.51	0.31	1.70	0.40	1.46	0.28
		SL + NLLLT	1.50	0.24	1.69	0.34	1.45	0.21
		SL + LLLT	1.54	0.33	1.69	0.46	1.50	0.29
Handicap	Lack of self-confidence	Conv. + NLLLT	3.45	0.37	3.55	0.37	2.45	0.37
		Conv. + LLLT	3.35	0.88	3.44	0.91	2.48	0.59
		SL + NLLLT	3.11	1.09	3.19	1.14	2.36	0.66
		SL + LLLT	3.10	1.16	3.18	1.20	2.35	0.75
	Difficulties in cleaning	Conv. + NLLLT	1.65	0.37	3.05	0.37	2.25	0.37
		Conv. + LLLT	1.78	0.43	3.00	0.76	2.30	0.54
		SL + NLLLT	1.76	0.36	2.81	0.92	2.21	0.58
		SL + LLLT	1.75	0.49	2.80	0.99	2.20	0.68

## Data Availability

All data are available within the manuscript in the form of tables and figures. Raw data can be made available upon reasonable request.
